# Palladium(II)/Cationic 2,2’-Bipyridyl System as a Highly Efficient and Reusable Catalyst for the Mizoroki-Heck Reaction in Water

**DOI:** 10.3390/molecules15010315

**Published:** 2010-01-12

**Authors:** Shao-Hsien Huang, Jun-Rong Chen, Fu-Yu Tsai

**Affiliations:** Institute of Organic and Polymeric Materials, National Taipei University of Technology, 1, Section 3, Chung-Hsiao E. Rd., Taipei 106, Taiwan

**Keywords:** Mizoroki-Heck reaction, water-soluble catalyst, reusable, cross-coupling

## Abstract

A water-soluble and air-stable Pd(NH_3_)_2_Cl_2_/cationic 2,2’-bipyridyl system was found to be a highly-efficient and reusable catalyst for the coupling of aryl iodides and alkenes in neat water using Bu_3_N as a base. The reaction was conducted at 140 °C in a sealed tube in air with a catalyst loading as low as 0.0001 mol % for the coupling of activated aryl iodides with butyl and ethyl acrylates, providing the corresponding products in good to excellent yields with very high turnover numbers. In the case of styrene, Mizoroki-Heck coupling products were obtained in good to high yields by using a greater catalyst loading (1 mol %) and TBAB as a phase-transfer agent. After extraction, the residual aqueous solution could be reused several times with only a slight decrease in its activity, making the Mizoroki-Heck reaction “greener”.

## 1. Introduction

Palladium-catalyzed coupling of aryl halides and alkenes, known as the Mizoroki-Heck reaction, is one of the most powerful processes available in organic synthesis for carbon–carbon bond formation [1,2]. This reaction is greatly facilitated in polar aprotic solvents such as DMA, DMF, DMSO, MeCN, and NMP under homogeneous catalysis (for recent reviews, see refs. [3,4,5,6]). However, the catalyst and organic products are often of similar solubility in organic solvents, and hence it is difficult to separate the catalyst from the reaction mixture and recycle it at the end of the reaction, leading to wastage of precious metals. Because water is an organic immiscible solvent, the introduction of a water-soluble ligand to combine with transition-metal catalysts and use of water as the reaction medium is one of the best methods of overcoming this problem (for recent reviews, see refs. [7,8,9,10,11,12,13,14]), although the reaction rates of organic reactions in water may be slower than in organic solvents due to the low effective concentration of the organic substrate in the aqueous phase. The water-soluble catalytic system may be easily separated from the water-insoluble organic products by simple filtration or extraction, leading to the possibility of reuse of the catalyst. Therefore, the development of a water-compatible and reusable catalytic system is highly attractive and valuable from the green chemistry and economics viewpoints.

**Scheme 1:**
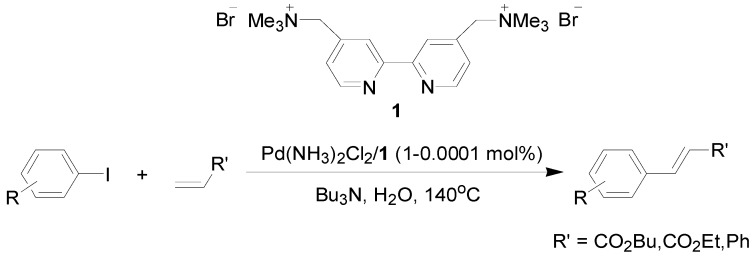
The Mizoroki-Heck reaction in water.

There are some reports of the Mizoroki-Heck reaction being performed in neat water, which include reactions in the presence of phase-transfer agents [15,16,17,18,19] and the use of hydrophilic ligands such as phosphines [20,21], oxime derivatives [22], oligoether-substituted benzimidazolim salts [23] and anionic *N*-donor ligands [24]. Other methods involving the use of supported materials [25,26,27,28,29,30,31] and Pd-nanoparticles [32,33,34] as heterogeneous catalysts also allow the reaction to be conducted in the aqueous phase. We have recently prepared water-soluble cationic 2,2’-bipyridyl ligand **1** and utilized it to bring a palladium complex into the aqueous phase for carbon–carbon bond-forming reactions [35,36,37], a rhodium complex for phenylacetylene polymerization [38], and an iron salt for *S*-arylation [39] under aerobic conditions. As part of our continuing efforts in the development of green and reusable catalytic systems for carbon–carbon bond-forming reactions, we report herein the combination of Pd(NH_3_)_2_Cl_2_ and **1** to create a highly-efficient and reusable catalyst for the coupling of aryl iodides and alkenes in water under air, making the Mizoroki-Heck reaction green and economically viable (Scheme 1).

## 2. Results and Discussion

The catalytic system was prepared by mixing equimolar amounts of Pd(NH_3_)_2_Cl_2_ and **1** in water. The catalyst is stable in water and can be stored under air; hence, stock solutions of this catalytic system could be prepared at different concentrations. In preliminary studies, our goal was to find the best base for use in the Mizoroki-Heck reaction in water (Table 1). We found that inorganic bases were ineffective for the coupling of iodobenzene **2a** and butyl acrylate **3a**, even at a reaction temperature of 140 °C (Entries 1–6). Replacing inorganic bases by organic amines such as Et_3_N led to the formation of *trans*-cinnamic acid *n*-butyl ester **4a** in a 32% yield (Entry 7). Among the amines used, the use of 2 equiv. of Bu_3_N provided the best results, and a near quantitative yield was obtained (Entry 10). The blank reaction was also examined under identical conditions, and no desired product was found in the absence of the catalyst, which indicates that the reaction is indeed catalyzed by the Pd system at a very low catalytic concentration. In the absence of **1**, a yield of only 34% was obtained, suggesting the importance of the use of a water-soluble ligand for reaction in water. Thus, the optimal reaction conditions were obtained and are shown in entry 10 of Table 1.

**Table 1:** Base screening for the Mizoroki-Heck reaction of iodobenzene **2a** and butyl acrylate **3a** in water.^a^

Entry	Base (equiv.)	Yield (%)^b^
1	NaOH (2)	NR
2	NaHCO_3_ (2)	2
3	KOH (2)	NR
4	KOAc (2)	NR
5	K_2_CO_3_ (2)	NR
6	KF (2)	4
7	Et_3_N (2)	32
8	Diisopropylamine (2)	17
9	*N,N*-Diisopropylethylamine (2)	6
10	Bu_3_N (2)	99
11	Bu_3_N (1)	49
12^c^	Bu_3_N (2)	NR
13^d^	Bu_3_N (2)	34

*^a ^Reaction conditions*: iodobenzene (1 mmol), butyl acrylate (1.5 mmol), base, Pd(NH_3_)_2_Cl/**1** (0.01 mol %), and H_2_O (3 mL) in 140 °C for 12 h; ^b ^Isolated yields; ^c ^In the absence of catalyst; ^d ^In the absence of **1.**

Using the optimal conditions, we next investigated the scope of the reaction of aryl iodides with butyl acrylate (Table 2). Iodobenzene and its derivatives with electron-withdrawing groups at the *para*-position afforded very high activities, offering the corresponding products, **4b–4f**, in excellent yields (Entries 2, 4, 6, 8, and 10, respectively). Further reduction of the catalyst loading to 0.0001 mol % gave the products at yields between 48% and 92% (Entry 1, 3, 5, 7, 9, and 11), and the turnover number (TON) was up to 920,000 (Entry 3). A longer reaction time was required for aryl iodides bearing an electron-donating group at the *para*-position. Hence, good to high yields could be isolated in 48 h with a catalyst loading of 0.01 mol % (Entries 12–15). Similarly, in the cases of entries 12–15, both *meta*- and *ortho*-substituted aryl iodides gave excellent isolated yields under identical conditions (Entries 16–18).

Although the boiling point of ethyl acrylate **3b** is lower than the reaction temperature, the Mizoroki-Heck coupling of aryl iodides with **3b** still proceeded smoothly in a sealed tube. As shown in Table 3, the corresponding products were isolated in yields slightly lower than that of the coupling with butyl acrylate under similar conditions (Entries 1–17). The catalyst loading for the reaction of activated aryl iodides with **3b** could also be further reduced to 0.0001 mol %, which led to the formation of the desired products in good to high yields with a very high TON (Entries 2, 4, 6, 8, 10, and 12).

**Table 2:** Mizoroki-Heck reaction of aryl iodides **2a-l** and butyl acrylate **3a** in water.^a^

Entry	Aryl iodide		Pd/1 (mol %)	Time (h)	Product (%)^b^		TON
1^c^	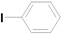	**2a**	0.0001	48	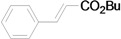	**4a** (48)	480,000
2	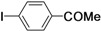	**2b**	0.01	12	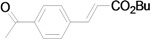	**4b** (99)	9,900
3^c^		**2b**	0.0001	48		**4b** (92)	920,000
4	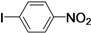	**2c**	0.01	12	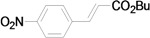	**4c** (99)	9,900
5^c^		**2c**	0.0001	48		**4c** (80)	800,000
6	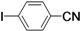	**2d**	0.01	12	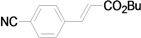	**4d** (99)	9,900
7^c^		**2d**	0.0001	48		**4d** (86)	860,000
8	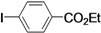	**2e**	0.01	12	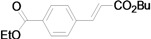	**4e** (91)	9,100
9^c^		**2e**	0.0001	48		**4e** (68)	680,000
10	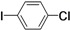	**2f**	0.01	12	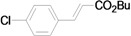	**4f** (92)	9,200
11^c^		**2f**	0.0001	48		**4f** (91)	910,000
12	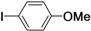	**2g**	0.01	12	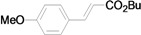	**4g** (67)	6,700
13		**2g**	0.01	48		**4g** (94)	9,400
14	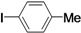	**2h**	0.01	48	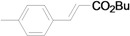	**4h** (92)	9,200
15	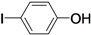	**2i**	0.01	48	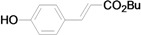	**4i** (56)	5,600
16	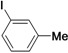	**2j**	0.01	48	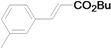	**4j** (96)	9,600
17	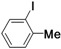	**2k**	0.01	48	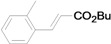	**4k** (95)	9,500
18	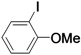	**2l**	0.01	48	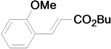	**4l** (96)	9,600

^a ^*Reaction conditions*: aryl iodide (1 mmol), butyl acrylate (1.5 mmol), Bu_3_N (2 mmol), Pd(NH_3_)_2_Cl/**1** (0.01 or 0.0001 mol %), and H_2_O (3 mL) at 140 °C; ^b ^Isolated yields. ^c ^Ten mmol of aryl iodide was used.

**Table 3:** Mizoroki-Heck reaction of aryl iodides **2a-k** and ethyl acrylate **3b** in water.^a^

Entry	Aryl iodide		Pd/1 (mol %)	Time (h)	Product (%)^b^		TON
1	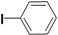	**2a**	0.01	12	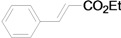	**5a** (63)	6,300
2^c^		**2a**	0.0001	48		**5a** (58)	580,000
3	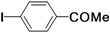	**2b**	0.01	12	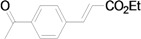	**5b** (82)	8,200
4^c^		**2b**	0.0001	48		**5b** (81)	810,000
5	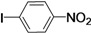	**2c**	0.01	12	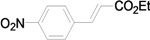	**5c** (74)	7,400
6^c^		**2c**	0.0001	48		**5c** (51)	510,000
7	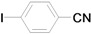	**2d**	0.01	12	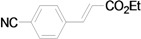	**5d** (80)	8,000
8^c^		**2d**	0.0001	48		**5d** (76)	760,000
9	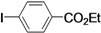	**2e**	0.01	12	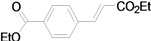	**5e** (81)	8,100
10^c^		**2e**	0.0001	48		**5e** (45)	450,000
11	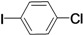	**2f**	0.01	12	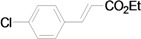	**5f** (82)	8,200
12^c^		**2f**	0.0001	48		**5f** (81)	810,000
13	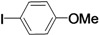	**2g**	0.01	48	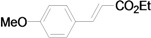	**5g** (82)	8,200
14	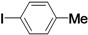	**2h**	0.01	48	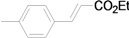	**5h** (74)	7,400
15	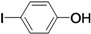	**2i**	0.01	48	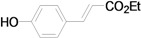	**5i** (44)	4,400
16	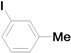	**2j**	0.01	48	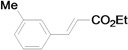	**5j** (59)	5,900
17	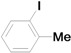	**2k**	0.01	48	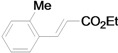	**5k** (65)	6,500

^a ^*Reaction conditions*: aryl iodide (1 mmol), ethyl acrylate (1.5 mmol), Bu_3_N (2 mmol), Pd(NH_3_)_2_Cl/**1** (0.01 or 0.0001 mol %), and H_2_O (3 mL) at 140 °C; ^b ^Isolated yields; ^c^ Ten mmol of aryl iodide was used.

The reaction rate of iodobenzene with styrene under this catalytic system was much slower than that of butyl and ethyl acrylate, providing only 36% of **6a** when 1 mol % of catalyst was employed (Table 4, Entry 1). The addition of a phase-transfer agent, TBAB, to the mixture improved the reaction efficiently to give **6a** in an 89% yield (Entry 2). Thus, TBAB was added when styrene was used as the reactant. Under such conditions, aryl iodides with not only electron-withdrawing but also electron-donating groups at the *para*-position could couple with styrene efficiently to afford the corresponding *trans*-stilbene derivatives, **6b–6i**, in high yields (Entries 3–9). In addition, 3-iodotoluene could also react with styrene to furnish **6j** in a 68% yield (Entry 10), and even sterically-hindered aryl iodides, **2k–2m**, were able to be coupled efficiently under this system, the corresponding products being obtained at yields between 73% and 93% at 140 °C within 24 h (Entries 11–13). 

**Table 4:** Mizoroki-Heck reaction of aryl iodides **2a-m** and styrene **3c** in water.^a^

Entry	Aryl iodide		Pd/1 (mol %)	TBAB (equiv)	Product (%)^b^	
1	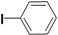	**2a**	1	0	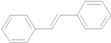	**6a** (36)
2		**2a**	1	1		**6a** (89)
3	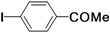	**2b**	1	1	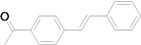	**6b** (93)
4	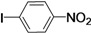	**2c**	1	1	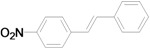	**6c** (82)
5	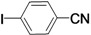	**2d**	1	1	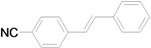	**6d** (83)
6	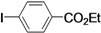	**2e**	1	1	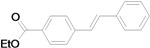	**6e** (76)
7	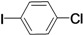	**2f**	1	1	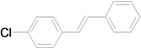	**6f** (87)
8	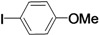	**2g**	1	1	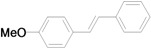	**6g** (82)
9	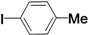	**2h**	1	1	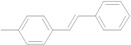	**6h** (71)
10	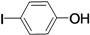	**2i**	1	1	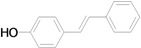	**6i** (69)
11	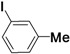	**2j**	1	1	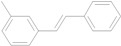	**6j** (68)
12	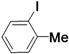	**2k**	1	1	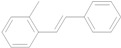	**6k** (73)
13	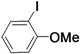	**2l**	1	1	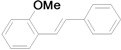	**6l** (93)
14	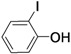	**2m**	1	1	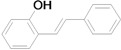	**6m** (84)

^a ^*Reaction conditions*: aryl iodide (1 mmol), styrene (1.5 mmol), Bu_3_N (2 mmol), Pd(NH_3_)_2_Cl/**1** (1 mol %), TBAB (1 mmol), and H_2_O (3 mL) at 140 °C for 24h; ^b ^Isolated yields.

**Scheme 2:**
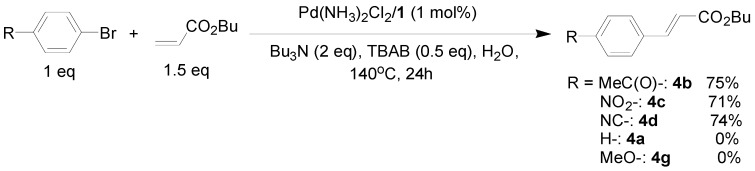
The Mizoroki-Heck reaction of aryl bromides with butyl acrylate in water.

As for the coupling of cheaper aryl bromides, we found that only activated aryl bromides could couple with butyl acrylate to afford the corresponding Mizoroki-Heck coupling products in good yields with 1 mol % catalyst in the presence of 0.5 equiv TBAB. The use of bromobenzene and a deactivated aryl bromide such as 4-bromoanisole did not furnish any desired product (Scheme 2).

We then examined the reusability of the residual aqueous solution, which is important from the viewpoints of practical utilization and economics. We employed butyl and ethyl acrylates and styrene individually to react with 4-iodoacetophone, **2b**, to test the reusability of this catalytic system (Table 5). The Mizoroki-Heck coupling of butyl acrylate and **2b** with a 0.01 mol % catalyst loading led to the formation of **4b** in a 99% yield in 12 h. After completion of the first cycle, hexane was used to extract the organic product and the residual aqueous solution was recharged with Bu_3_N, butyl acrylate, and 4-iodoacetophone for the second cycle. It was found that the residual aqueous solution could be reused at least four times with only a slight decrease in activity (Entry 1). The employment of ethyl acrylate led to a similar reusability with slightly lower yields as compared with butyl acrylate (Entry 2). In the case of styrene, a higher catalyst loading (1 mol %) and TBAB were employed in the initial cycle in order to lead to a higher product yield. Addition of the phase-transfer agent in subsequent runs was not required (Entry 3).

**Table 5:** Reuse studies of the Mizoroki-Heck reaction of 4-iodoacetophone **2b** and alkenes **3**.^a^

Entry	Alkene		Time (h)	Pd/1 (mol %)	Cycle (%)^b^				
					1st	2nd	3rd	4th	5th
1	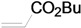	**3a**	12	0.01	99	98	95	93	90
2	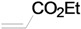	**3b**	12	0.01	90	90	86	83	81
3^c^	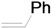	**3c**	24	1	93	94	93	92	90

^a ^*Reaction conditions*: 4-iodoacetophone (1 mmol), alkene (1.5 mmol), Bu_3_N (2 mmol), Pd(NH_3_)_2_Cl/**1** (1 or 0.01 mol %), and H_2_O (3 mL) at 140 °C; ^b ^Isolated yields; ^c^ One mmol of TBAB was added.

## 3. Experimental

### 3.1. General

Chemicals were purchased from commercial suppliers and were used without further purification. The cationic 2,2’-bipyridyl ligand was prepared according to the published procedures [24,25]. GC analysis was performed on a Shimadzu GC-14B equipped with a fused silica capillary column, and all ^1^H- and ^13^C-NMR spectra were recorded in CDCl_3_ at 25 °C on a Varian 200 NMR spectrometer. Elemental analyses were performed and high resolution mass spectra recorded at the Instrument Center Service, National Science Council of Taiwan. 

### 3.2. Typical procedure for the Mizoroki-Heck reaction

A sealable tube equipped with a magnetic stirrer bar was charged with aryl iodide (1 mmol), alkene (1.5 mmol), Bu_3_N (2 mmol), and H_2_O (2 mL). In the case of styrene, the addition of TBAB (1 mmol) was required. After the addition of Pd(NH_3_)_2_Cl_2_/**1** aqueous solution (in 1 mL H_2_O, at different concentrations for various substrate/catalyst ratios), the tube was sealed under air by a Teflon-coated screw cap. The reaction vessel was then placed in an oil bath at 140 °C for the indicated reaction time period (see Table 2, Table 3 and Table 4). After cooling of the reaction mixture to room temperature, the aqueous solution was extracted with hexane, the organic phase was dried over MgSO_4_, and the solvent was then removed under vacuum. Column chromatography on silica gel afforded the desired product.

*(E)-3-Phenylacrylic* *acid* *n-butyl ester* (**4a**) [40]. Yellow oil. ^1^H-NMR: δ 0.97 (t, *J* = 7.0 Hz, 3H), 1.35–1.53 (m, 2H), 1.63–1.77 (m, 2H), 4.21 (t, *J* = 6.6 Hz, 2H), 6.44 (d, *J* = 16.0 Hz, 1H), 7.35–7.41 (m, 3H), 7.49–7.56 (m, 2H), 7.67 (d, *J* = 16.0 Hz, 1H); ^13^C NMR: δ 13.6, 19.1, 30.7, 64.2, 118.2, 127.8, 128.7, 129.9, 134.4, 144.3, 166.8.

*(E)-3-(4-Acetylphenyl)acrylic acid n-butyl ester* (**4b**) [41]. Yellow oil. ^1^H-NMR: δ 0.94 (t, *J* = 7.6 Hz, 3H), 1.34–1.51 (m, 2H), 1.61–1.75 (m, 2H), 2.60 (s, 3H), 4.21 (t, *J* = 6.4 Hz, 2H), 6.51 (d, *J* = 16.0 Hz, 1H), 7.59 (d, *J* = 8.0 Hz, 2H), 7.67 (d, *J* = 16.0 Hz, 1H), 7.95 (d, *J* = 8.0 Hz, 2H); ^13^C-NMR: δ 13.6, 19.1, 26.5, 30.7, 64.5, 120.7, 128.0, 128.7, 137.9, 138.7, 142.8, 166.3, 197.0.

*(E)-3-(4-Nitrophenyl)acrylic acid n-butyl ester* (**4c**). Yellow solid. Mp. 67–68 (lit.[41] 67–69 °C). ^1^H-NMR: δ 0.95 (t, *J* = 7.4 Hz, 3H), 1.33–1.51 (m, 2H), 1.61–1.76 (m, 2H), 4.22 (t, *J* = 6.6 Hz, 2H), 6.54 (d, *J* = 16.0 Hz, 1H), 7.62–7.73 (m, 3H), 8.23 (d, *J* = 9.0 Hz, 2H); ^13^C-NMR: δ 13.6, 19.1, 30.7, 64.8, 122.6, 124.0, 128.5, 140.5, 141.4, 148.4, 165.9.

*(E)-3-(4-Cyanophenyl)acrylic acid n-butyl ester* (**4d**). Yellow solid. Mp. 43.1–43.5 (lit.[42] 43.5–46.9 °C). ^1^H-NMR: δ 0.95 (t, *J* = 7.2 Hz, 3H), 1.32–1.51 (m, 2H), 1.61–1.75 (m, 2H), 4.21 (t, *J* = 6.6 Hz, 2H), 6.50 (d, *J* = 16.2 Hz, 1H), 7.56–7.68 (m, 5H); ^13^C-NMR: δ 13.5, 19.0, 30.5, 64.5, 113.1, 118.1, 121.7, 128.2, 132.4, 138.5, 141.8, 165.9.

*(E)-4-(2-Butoxycarbonylvinyl)benzoic acid ethyl ester* (**4e**). Yellow oil. ^1^H-NMR: δ 0.97 (t, *J* = 7.2 Hz, 3H), 1.36–1.57 (m, 5H), 1.63–1.77 (m, 2H), 4.22 (t, *J* = 6.6 Hz, 2H), 4.39 (q, *J* = 7.2 Hz, 2H), 6.52 (d, *J* = 16.0 Hz, 1H), 7.58 (d, *J* = 8.4 Hz, 2H), 7.69 (d, *J* = 16.0 Hz, 1H), 8.06 (d, *J* = 8.4 Hz, 2H); ^13^C- NMR: δ 13.5, 14.1, 18.9, 30.5, 60.9, 64.3, 120.4, 127.5, 129.7, 131.4, 138.3, 142.8, 165.5, 166.1; HRMS calcd. for C_16_H_20_O_4_, 276.1362; found, 276.1353.

*(E)-3-(4-Chlorophenyl)acrylic acid n-butyl ester* (**4f**) [43]. Colorless oil. ^1^H-NMR: δ 0.96 (t, *J* = 7.4 Hz, 3H), 1.34–1.53 (m, 2H), 1.62–1.76 (m, 2H), 4.21 (t, *J* = 6.6 Hz, 2H), 6.41 (d, *J* = 16.0 Hz, 1H), 7.35 (d, *J* = 8.6 Hz, 2H), 7.46 (d, *J* = 8.6 Hz, 2H), 7.62 (d, *J* = 16.0 Hz, 1H); ^13^C-NMR: δ 13.5, 19.9, 30.6, 64.2, 188.7, 128.8, 128.9, 132.8, 135.8, 142.7, 166.3.

*(E)-3-(4-Methoxyphenyl)acrylic acid n-butyl ester* (**4g**) [40]. Yellow oil. ^1^H-NMR: δ 0.96 (t, *J* = 7.2 Hz, 3H), 1.26–1.53 (m, 2H), 1.62–1.76 (m, 2H), 3.84 (s, 3H), 4.20 (t, *J* = 6.4 Hz, 2H), 6.31 (d, *J* = 16.0 Hz, 1H), 6.90 (d, *J* = 8.6 Hz, 2H), 7.48 (d, *J* = 8.6 Hz, 2H), 7.64 (d, *J* = 16.0 Hz, 1H); ^13^C-NMR: δ 13.7, 19.1, 30.8, 55.2, 64.1, 114.2, 115.7, 127.1, 129.5, 144.1, 161.2, 167.2.

*(E)-3-(4-Tolyl)acrylic acid n-butyl ester* (**4h**) [41]. Yellow oil. ^1^H-NMR: δ 0.96 (t, *J* = 7.2 Hz, 3H), 1.38–1.53 (m, 2H), 1.62–1.76 (m, 2H), 2.37 (s, 3H), 4.20 (t, *J* = 6.6 Hz, 2H), 6.39 (d, *J* = 16.0 Hz, 1H), 7.19 (d, *J* = 8.0 Hz, 2H), 7.43 (d, *J* = 8.0 Hz, 2H), 7.66 (d, *J* = 16.0 Hz, 1H); ^13^C-NMR: δ 13.5, 19.0, 21.2, 30.7, 64.0, 117.1, 127.8, 129.3, 131.6, 140.3, 144.2, 166.9.

*(E)-3-(4-Hydroxyphenyl)acrylic acid n-butyl ester* (**4i**). Yellow solid. Mp. 74–76 (lit.[44] 72–74 °C). ^1^H-NMR: δ 0.94 (t, *J* = 7.2 Hz, 3H), 1.32–1.50 (m, 2H), 1.63–1.74 (m, 2H), 4.18 (t, *J* = 6.4 Hz, 2H), 5.29 (s, 1H), 6.28 (d, *J* = 16.0 Hz, 1H), 6.82 (d, *J* = 8.6 Hz, 2H), 7.42 (d, *J* = 8.6 Hz, 2H), 7.61 (d, *J* = 16.0 Hz, 1H); ^13^C-NMR: δ 13.6, 19.1, 30.6, 64.6, 114.5, 116.0, 126.2, 129.9, 145.2, 158.9, 168.5.

*(E)-3-(3-Tolyl)acrylic acid n-butyl ester* (**4j**) [41]. Colorless oil. ^1^H-NMR: δ 0.97 (t, *J* = 7.6 Hz, 3H), 1.38–1.54 (m, 2H), 1.62–1.73 (m, 2H), 2.37 (s, 3H), 4.21 (t, *J* = 6.8 Hz, 2H), 6.42 (d, *J* = 16.0 Hz, 1H), 7.18–7.35 (m, 4H), 7.65 (d, *J* = 16.0 Hz, 1H); ^13^C-NMR: δ 13.5, 19.0, 21.0, 30.7, 64.0, 117.9, 124.9, 128.4, 128.4, 130.7, 134.2, 138.1, 144.4, 166.7.

*(E)-3-(2-Tolyl)acrylic acid n-butyl ester* (**4k**) [45]. Pale yellow oil. ^1^H-NMR: δ 0.97 (t, *J* = 7.2 Hz, 3H), 1.38–1.54 (m, 2H), 1.63–1.74 (m, 2H), 2.44 (s, 3H), 4.22 (t, *J* = 6.8 Hz, 2H), 6.36 (d, *J* = 16.0 Hz, 1H), 7.21 (m, 3H), 7.55 (d, *J* = 7.0 Hz, 1H), 7.98 (d, *J* = 16.0 Hz, 1H); ^13^C-NMR: δ 13.7, 19.2, 19.7, 30.8, 64.4, 119.3, 126.2, 126.4, 129.8, 130.7, 133.4, 137.5, 142.2, 167.0.

*(E)-3-(2-Methoxyphenyl)acrylic acid n-butyl ester* (**4l**) [46]. Yellow oil. ^1^H-NMR: δ 0.94 (t, *J* = 7.4 Hz, 3H), 1.36–1.51 (m, 2H), 1.61–1.74 (m, 2H), 3.87 (s, 3H), 4.19 (t, *J* = 6.6 Hz, 2H), 6.51 (d, *J* = 16.0 Hz, 1H), 6.88–6.98 (m, 2H), 7.28–7.36 (m, 1H), 7.49 (d, *J* = 7.8 Hz, 1H), 7.97 (d, *J* = 16.2 Hz, 1H); ^13^C-NMR: δ 13.5, 19.0, 30.7, 55.1, 63.9, 110.9, 118.5, 120.4, 123.2, 128.5, 131.1, 139.6, 158.0, 167.2.

*(E)-3-Phenylacrylic* *acid ethyl ester* (**5a**) [47]. Oil. ^1^H-NMR: δ 1.32 (t, *J* = 7.2 Hz, 3H), 4.25 (q, *J* = 7.2 Hz, 2H), 6.42 (d, *J* = 16.0 Hz, 1H), 7.35–7.38 (m, 3H), 7.49–7.53 (m, 2H), 7.67 (d, *J* = 16.0 Hz, 1H); ^13^C-NMR: δ 14.2, 60.3, 118.2, 127.9, 128.7, 130.0, 134.4, 144.4, 166.8.

*(E)-3-(4-Acetylphenyl)acrylic acid ethyl ester* (**5b**). Yellow solid. Mp. 43–45 °C (lit.[48] 40–42 °C). ^1^H-NMR: δ 1.35 (t, *J* = 7.2 Hz, 3H), 2.62 (s, 3H), 4.28 (q, *J* = 7.2 Hz, 2H), 6.53 (d, *J* = 16.0 Hz, 1H), 7.61 (d, *J* = 8.4 Hz, 2H), 7.70 (d, *J* = 16.0 Hz, 1H), 7.97 (d, *J* = 8.4 Hz, 2H); ^13^C-NMR: δ 14.0, 26.3, 60.4, 120.5, 127.7, 128.5, 137.6, 138.4, 142.6, 165.9, 196.7.

*(E)-3-(4-Nitrophenyl)acrylic acid ethyl ester* (**5c**). Yellow solid. Mp. 134–136 °C (lit.[49] 132–134 °C). ^1^H-NMR: δ 1.36 (t, *J* = 7.2 Hz, 3H), 4.29 (q, *J* = 7.2 Hz, 2H), 6.56 (d, *J* = 16.0 Hz, 1H), 7.67 (d, *J* = 8.8 Hz, 2H), 7.71 (d, *J* = 16.0 Hz, 1H), 8.25 (d, *J* = 8.8 Hz, 2H); ^13^C-NMR: δ 14.2, 60.9, 122.5, 124.0, 128.5, 140.5, 141.4, 148.4, 165.8.

*(E)-3-(4-Cyanophenyl)acrylic acid ethyl ester* (**5d**). White solid. Mp. 68–70 °C (lit.[50] 69–69.3 °C). ^1^H-NMR: δ1.32 (t, *J* = 7.2 Hz, 3H), 4.26 (q, *J* = 7.2 Hz, 2H), 6.49 (d, *J* = 16.0 Hz, 1H), 7.56–7.68 (m, 5H); ^13^C-NMR: δ 14.2, 60.9, 113.3, 118.2, 121.9, 128.2, 132.5, 138.7, 142.0, 165.9.

*(E)-4-(2-Ethoxycarbonylvinyl)benzoic acid ethyl ester* (**5e**). Yellow solid. Mp. 51–53 °C. ^1^H-NMR: δ 1.35 (t, *J* = 7.2 Hz, 3H), 1.40 (t, *J* = 7.0 Hz, 3H), 4.28 (q, *J* = 7.2 Hz, 2H), 4.39 (q, *J* = 7.0 Hz, 2H), 6.52 (d, *J* = 16.0 Hz, 1H), 7.58 (d, *J* = 8.6 Hz, 2H), 7.70 (d, *J* = 16.0 Hz, 1H), 8.05 (d, *J* = 8.6 Hz, 2H); ^13^C-NMR: δ 14.2, 60.6, 61.0, 120.5, 127.7, 129.9, 131.6, 138.4, 143.0, 165.7, 166.3; Anal. calcd. for C_14_H_16_O_4_: C, 67.73; H, 6.50, found C, 67.91; H, 6.64.

*(E)-3-(4-Chlorophenyl)acrylic acid ethyl ester* (**5f**) [51]. Oil. ^1^H-NMR: δ 1.34 (t, *J* = 7.2 Hz, 3H), 4.27 (q, *J* = 7.2 Hz, 2H), 6.41 (d, *J* = 16.0 Hz, 1H), 7.35 (d, *J* = 8.6 Hz, 2H), 7.46 (d, *J* = 8.6 Hz, 2H), 7.63 (d, *J* = 16.0 Hz, 1H); ^13^C-NMR: δ 14.3, 60.5, 118.8, 128.6, 129.1, 132.9, 136.0, 142.9, 166.6.

*(E)-3-(4-Methoxyphenyl)acrylic acid ethyl ester* (**5g**) [51]. Oil. ^1^H-NMR: δ 1.33 (t, *J* = 7.2 Hz, 3H), 3.84 (s, 3H), 4.25 (q, *J* = 7.2 Hz, 2H), 6.31 (d, *J* = 16.0 Hz, 1H), 6.90 (d, *J* = 8.6 Hz, 2H), 7.48 (d, *J* = 8.6 Hz, 2H), 7.64 (d, *J* = 16.0 Hz, 1H); ^13^C-NMR: δ 14.1, 55.0, 60.0, 114.0, 115.5, 126.9, 129.4, 143.9, 161.1, 166.9.

*(E)-3-(4-Tolyl)acrylic acid ethyl ester* (**5h**) [51]. Oil. ^1^H-NMR: δ 1.34 (t, *J* = 7.2 Hz, 3H), 2.37 (s, 3H), 4.26 (q, *J* = 7.2 Hz, 2H), 6.39 (d, *J* = 16.0 Hz, 1H), 7.19 (d, *J* = 8.0 Hz, 2H), 7.43 (d, *J* = 8.0 Hz, 2H), 7.66 (d, *J* = 16.0 Hz, 1H); ^13^C-NMR: δ 14.2, 21.3, 60.2, 117.1, 127.9, 129.4, 131.6, 140.4, 144.4, 166.9.

*(E)-3-(4-Hydroxyphenyl)acrylic acid ethyl ester* (**5i**) [52]. Oil. ^1^H-NMR: δ 1.33 (t, *J* = 7.2 Hz, 3H), 4.25 (q, *J* = 7.2 Hz, 2H), 6.29 (d, *J* = 15.8 Hz, 1H), 6.84 (d, *J* = 8.4 Hz, 2H), 7.43 (d, *J* = 8.4 Hz, 2H), 7.63 (d, *J* = 15.8 Hz, 1H); ^13^C-NMR: δ 14.3, 60.4, 114.7, 116.1, 126.1, 129.9, 144.9, 159.2, 167.8.

*(E)-3-(3-Tolyl)acrylic acid ethyl ester* (**5j**) [53]. Oil. ^1^H-NMR: δ 1.34 (t, *J* = 7.2 Hz, 3H), 2.37 (s, 3H), 4.26 (q, *J* = 7.2 Hz, 2H), 6.42 (d, *J* = 16.0 Hz, 1H), 7.17–7.34 (m, 4H), 7.66 (d, *J* = 16.0 Hz, 1H); ^13^C-NMR: δ 14.2, 21.2, 60.3, 117.9, 125.1, 128.6, 128.6, 130.9, 134.3, 138.3, 144.6, 166.8.

*(E)-3-(2-Tolyl)acrylic acid ethyl ester* (**5k**) [51]. Oil. ^1^H-NMR: δ 1.35 (t, *J* = 7.2 Hz, 3H), 2.45 (s, 3H), 4.27 (q, *J* = 7.2 Hz, 2H), 6.36 (d, *J* = 16.0 Hz, 1H), 7.21–7.26 (m, 3H), 7.55 (d, *J* = 7.2 Hz, 1H), 7.98 (d, *J* = 16.0 Hz, 1H); ^13^C-NMR: δ 14.2, 19.6, 60.3, 119.2, 126, 2, 126.3, 129.8, 130.6, 133.3, 137.4, 142.1, 166.8.

*(E)-Stilbene* (**6a**). Yellow solid. Mp. 125–126 °C (lit.[54] 125 °C). ^1^H-NMR: δ 7.11 (s, 2H), 7.29 (t, *J* = 6.6 Hz, 2H), 7.40 (t, *J* = 7.0 Hz, 4H), 7.53 (d, *J* = 8.2 Hz, 4H); ^13^C-NMR: δ 126.5, 127.6, 128.6, 128.7, 137.3.

*(E)-4-Acetylstilbene* (**6b**). White solid. Mp. 145–147 °C (lit.[55] 148–150 °C). ^1^H-NMR: δ 2.61 (s, 3H), 7.12 (d, *J* = 16.4 Hz, 1H), 7.23 (d, *J* = 16.4 Hz, 1H), 7.26–7.43 (m, 3H), 7.55 (d, *J* = 8.0 Hz, 2H), 7.59 (d, *J* = 8.4 Hz, 2H), 7.95 (d, *J* = 8.4 Hz, 2H); ^13^C-NMR: δ 26.6, 126.4, 126.8, 127.4, 128.3, 128.7, 128.8, 131.4, 135.9, 136.7, 141.9, 197.3.

*(E)-4-Nitrostilbene* (**6c**). Yellow solid. Mp. 158–160 °C (lit.[56] 158–158.5 °C). ^1^H-NMR: δ 7.14 (d, *J* = 16.2 Hz, 1H), 7.28 (d, *J* = 16.2 Hz, 1H), 7.34–7.45 (m, 3H), 7.55 (d, *J* = 8.0 Hz, 2H), 7.64 (d, *J* = 8.0 Hz, 2H); ^13^C-NMR: δ 124.1, 126.3, 126.8, 126.9, 128.7, 128.8, 133.3, 136.2, 143.8, 146.8.

*(E)-4-Cyanostilbene* (**6d**). White solid. Mp. 120 °C (lit.[55] 117.4–117.7 °C). ^1^H-NMR: δ 7.08 (d, *J* = 16.0 Hz, 1H), 7.22 (d, *J* = 16.0 Hz, 1H), 7.32–7.44 (m, 3H), 7.52–7.67 (m, 6H); ^13^C-NMR: δ 110.6, 118.9, 126.7, 126.8, 126.9, 128.6, 128.8, 132.3, 132.4, 136.3, 141.8.

*(E)-4-Ethoxycarbonylstilbene* (**6e**). Colorless solid. Mp 105–107 °C (lit.[57] 106.0–106.5 °C). ^1^H-NMR: δ 1.41(t, *J* = 7.2 Hz, 3H), 4.39(q, *J* = 7.2 Hz, 2H), 7.12 (d, *J* = 16.2 Hz, 1H), 7.23 (d, *J* = 16.2 Hz, 1H), 7.29–7.33 (m, 1H), 7.34–7.43 (m, 2H), 7.53–7.59 (m, 4H), 8.03 (d, *J* = 8.4 Hz, 2H); ^13^C- NMR: δ 14.8, 60.9, 126.2, 126.7, 127.6, 128.2, 128.7, 129.3, 129.9, 131.1, 136.7, 141.7, 166.3.

*(E)-4-Chlorostilbene* (**6f**). White solid. Mp. 130–132 °C (lit.[58] 129 °C). ^1^H-NMR: δ 7.07 (s, 2H), 7.26–7.41 (m, 5H), 7.44 (d, *J* = 8.8 Hz, 2H), 7.50 (d, *J* = 7.4 Hz, 2H); ^13^C-NMR: δ 126.5, 127.3, 127.6, 127.8, 128.7, 128.8, 129.3, 133.1, 135.8, 136.9.

*(E)-4-Methoxystilbene* (**6g**). Pale white solid. Mp. 135–136 °C (lit.[58] 136 °C). ^1^H-NMR: δ 3.82 (s, 3H), 6.89 (d, *J* = 8.8 Hz, 2H), 6.95 (d, *J* = 16.4 Hz, 1H), 7.07 (d, *J* = 16.4 Hz, 1H), 7.20–7.22 (m, 1H), 7.33 (t, *J* = 7.4 Hz, 2H), 7.41–7.50 (m, 4H); ^13^C-NMR: δ 55.3, 114.1, 126.2, 126.6, 127.2, 127.7, 128.2, 128.6, 130.2, 137.6, 159.3.

*(E)-4-Methylstilbene* (**6h**). Light yellow solid. Mp. 120–121 °C (lit.[58] 121 °C). ^1^H-NMR: δ 2.36 (s, 3H), 7.07 (s, 2H), 7.17 (d, *J* = 8.0 Hz, 2H), 7.24–7.26 (m, 1H), 7.35 (t, *J* = 7.4 Hz, 2H), 7.41 (d, *J* = 8.0 Hz, 2H), 7.50 (d, *J* = 7.2 Hz, 2H); ^13^C-NMR: δ 21.3, 126.3, 126.4, 127.4, 127.7, 128.6, 129.4, 134.5, 137.46, 137.49.

*(E)-4-Hydroxystilbene* (**6i**). Yellow solid. Mp. 185–187 °C (lit.[59] 186 °C). ^1^H-NMR: δ 4.78 (s, 1H), 6.83 (d, *J* = 8.8 Hz, 2H), 6.95 (d, *J* = 16.4 Hz, 1H), 7.06 (d, *J* = 16.4 Hz, 1H), 7.19–7.26 (m, 1H), 7.32 (d, *J* = 7.6 Hz, 2H), 7.41 (d, *J* = 8.6 Hz, 2H), 7.49 (d, *J* = 7.2 Hz, 2H); ^13^C-NMR: δ 115.6, 126.2, 126.7, 127.2, 127.9, 128.1, 128.6, 130.4, 137.6, 155.2.

*(E)-3-Methylstilbene* (**6j**). Pale yellow solid. Mp. 48–49 °C (lit.[60] 47–48 °C). ^1^H-NMR: δ 2.37 (s, 3H), 7.05–7.08 (m, 3H), 7.22–7.28 (m, 2H), 7.29–7.39 (m, 4H), 7.49–7.53 (m, 2H); ^13^C-NMR: δ 24.4, 123.7, 126.5, 127.2, 127.5, 128.4, 128.5, 128.5, 128.6, 128.8, 137.3, 137.4, 138.2.

*(E)-2-Methylstilbene* (**6k**) [16]. Oil. ^1^H-NMR: δ 2.42 (s, 3H), 6.99 (d, *J* = 16.2 Hz, 1H), 7.23–7.33 (m, 4H), 7.37–7.43 (m, 3H), 7.52 (d, *J* = 7.2 Hz, 2H), 7.59 (d, *J* = 6.8 Hz, 1H); ^13^C-NMR: δ 19.9, 125.4, 126.2, 126.5, 127.5, 127.6, 128.6, 129.9, 130.3, 135.7, 136.4, 137.7.

*(E)-2-Methoxystilbene* (**6l**). Pink solid. Mp. 58–59 °C (lit.[61] 58.6–59.5 °C). ^1^H-NMR: δ 3.89 (s, 3H), 6.91 (d, *J* = 8.4 Hz, 1H), 6.98 (d, *J* = 7.4 Hz, 1H), 7.11 (d, *J* = 16.6 Hz, 1H),7.26–7.43 (m, 5H), 7.55–7.62 (m, 3H); ^13^C NMR: δ 55.5, 110.9, 120.7, 123.5, 126.4, 126.44, 126.5, 127.3, 128.5, 128.6, 129.1, 137.9, 156.9.

*(E)-2-Hydroxystilbene* (**6m**). Pale white solid. Mp. 144–145 °C (lit.[62] 143–144 °C). ^1^H-NMR: δ 5.02 (s, 1H), 6.81 (dd, *J* = 1.6 Hz, *J* = 8.0 Hz, 1H), 6.95 (t, *J* = 7.4 Hz, 1H), 7.12 (d, *J* = 16.6 Hz, 1H), 7.16 (td, *J* = 1.6 Hz, *J* = 8.0 Hz, 1H), 7.21–7.29 (m, 1H), 7.37 (d, *J* = 16.6 Hz, 1H), 7.32–7.42 (m, 2H), 7.51–7.55 (m, 3H); ^13^C-NMR: δ 115.9, 121.1, 123.0, 124.7, 126.5, 127.3, 127.6, 128.6, 130.2, 137.6, 152.9.

### 3.3. Typical procedure for the reuse of the catalytic aqueous solution

The reaction was conducted following the procedure described in Section 3.2 under the reaction conditions shown in Table 5. After reaction, the aqueous reaction mixture was washed with hexane under vigorous stirring three times, and the organic product was isolated from the combined organic phase according to the previously-described procedure. The residual aqueous solution was then charged with aryl iodide, alkene, and Bu_3_N for the next reaction, and in the case of styrene, addition of TBAB at the first run was required.

## 4. Conclusions

In conclusion, we have shown here that the above-described Pd(NH_3_)_2_Cl_2_/cationic 2,2’-bipyridyl system could be a highly-efficient catalyst for the Mizoroki-Heck coupling of aryl iodides and alkenes using an environmentally benign solvent, water, as the reaction medium. The loading amount of the catalyst in a single batch reaction can be reduced to as low as 0.0001 mol %, while still affording the products in high yields. This water-compatible and air-stable catalytic system enables the reaction to be conducted using a very simple procedure. The catalyst can be easily separated from the organic products by simple extraction and the residual aqueous solution can be reused for further reactions, which reduces the wastage of precious metal, making this procedure greener and economically viable.
